# A Concentration Method for HIV Drug Resistance Testing in Low-Level Viremia Samples

**DOI:** 10.1155/2022/2100254

**Published:** 2022-11-23

**Authors:** Qun Li, Fengting Yu, Chuan Song, Hongxin Zhao, Liting Yan, Qing Xiao, Xiaojie Lao, Siyuan Yang, Yunxia Tang, Jiang Xiao, Fujie Zhang

**Affiliations:** ^1^Beijing Ditan Hospital, Capital Medical University, Beijing, China; ^2^Clinical Center for HIV/AIDS, Capital Medical University, Beijing, China; ^3^Beijing Key Laboratory of Emerging Infectious Diseases, Beijing, China

## Abstract

**Background:**

Drug resistance testing in HIV-1 low-level viremia (LLV) samples is challenging yet critical. Our study is aimed at assessing the performance of lentivirus concentration reagent (LCR) in combination with a validated Sanger sequencing (SS) for monitoring drug resistance mutations (DRMs) in LLV samples.

**Methods:**

A series of clinical samples were diluted and amplified for genotypic resistance testing (GRT) to prove the performance of the LCR. The Stanford HIV-1 drug resistance database (HIVdb version 8.9) was used to analyze the mutations. HIV-1 subtypes and CRFs were determined using the COMET online tool. The overall success rate of genotyping was compared with ultracentrifugation combined with SS. Furthermore, the success rates at varied VL of the two concentration methods were evaluated, and the DRMs of diluted samples were compared with those undiluted samples.

**Results:**

When LCR was used, the overall success rate was 90% (72/80) in the PR and RT regions and 60% (48/80) in the IN region. In addition, when HIV RNA was 1000 copies/ml, 400 copies/ml, 200 copies/ml, and 100 copies/ml, the success rates of PR and RT regions were 100%, 100%, 95%, and 65%, respectively, while the success rates of IN region were 85%, 60%, 45%, and 50%, respectively. We found that the sample DR-387A2 missed the E138A mutation, and mutations in other samples were consistent with undiluted samples using LCR.

**Conclusions:**

LCR will support monitoring DRMs in HIV-1 patients with LLV and can be an effective alternative for small- and medium-sized laboratories that cannot afford an ultracentrifuge.

## 1. Introduction

The growing knowledge has suggested the critical role of HIV-1 resistance genotyping testing (RGT) in drug resistance mutation (DRMs) monitoring [1]. Successful viral nucleic acid extraction and PCR amplification are critical steps for HIV-1 RGT. However, when the viral load (VL) of patients is low, such as low-level viremia (LLV), there is almost no available genomic template to use [2].

Previous studies illustrated that about 5.3%-38.7%% of patients could be detected as LLV in China [3–6]. LLV, which could lead to accumulation of DRMs [7, 8], virologic failure (VF) [4–6], and disease progression [9], was defined as VL between 50 and 999 copies/ml after six months of antiviral therapy (ART) according to World Health Organization (WHO) guidelines [10]. Solving the problem of drug resistance testing in LLV patients is very important for optimizing the ART regimen.

However, testing DRMs in HIV-1 patients with low VL is challenging, mainly because amplification of HIV segments in those patients is usually unsuccessful. Previous studies have improved the RGT success rate of LLV samples to 80%-90% by optimizing PCR amplification procedures or using magnetic silica extraction [2, 11–13]. An alternative strategy to address this problem is to use larger input plasma volumes for HIV RNA extraction [14]. In addition, a virus particle concentration step (ultracentrifugation) could be performed before the routine nucleic acid extraction procedure [7, 15]. Because of financial constraints, ultracentrifuges are unavailable for all laboratories in China or other low- and middle-income countries (LMICs). Hence, providing an alternative, cost-effective, and less laborious assay to concentrate virus is essential, which will be a great asset.

In the current study, we provided a commercially available lentiviral concentration reagent (LCR) to concentrate HIV particles and compared its performance with ultracentrifugation.

## 2. Materials and Methods

### 2.1. Selection of Patients

This retrospective single-centre study was conducted at Beijing Ditan Hospital from 22 March 2021 to 1 April 2022. In our study, 20 HIV-1 patients with at least one mutation and known subtypes were included, and their plasma samples were serially diluted to 1000, 400, 200, and 100 copies/ml by using sterile 1640 PBS buffer. In addition, every diluted samples were divided into two aliquots (2.1 ml of each), and 160 samples were obtained.

### 2.2. HIV-1 Drug Resistance Testing

#### 2.2.1. Enrichment Using Lentiviral Concentration Reagent

A4, A3, A2, and A1 represent samples diluted to 1000 copies/ml, 400 copies/ml, 200 copies/ml, and 100 copies/ml, respectively, concentrated by LCR. Add 0.7 ml of Buffer LP (ViraTrap™ Lentivirus Concentration Reagent ViraTrap™, Biomiga Medical Technology Co., Ltd.) to 2.1 ml diluted samples. Mix well and incubate overnight at 4°C. The virus can be stored in Buffer LP for 24 hours. Centrifuge the sample at 3,164 g for 30 minutes at 4°C. Carefully aspirate the supernatant. Spin briefly and remove the residual supernatant. The virus-containing pellet should be visible. Suspend the pellet with 200 *μ*l Buffer LS (ViraTrap™ Lentivirus Concentration Reagent ViraTrap™, Biomiga Medical Technology Co., Ltd.) and dissolve the pellet by pipetting. The suspension was transferred to PCR tubes and stored at –80°C until use.

LCR is based on the principle of physical precipitation. Buffer LP can combine with the HIV-1 virus and settle it to the bottom of the tube, but it does not damage the structure of HIV-1. The HIV-1 virus pellet was dissolved by adding buffer LS. HIV-1 was concentrated nearly 50–100 times through centrifugation and resuspension.

### 2.3. Enrichment by Ultracentrifugation

B4, B3, B2, and B1 represent samples diluted to 1000 copies/ml, 400 copies/ml, 200 copies/ml, and 100 copies/ml, respectively, concentrated by ultracentrifugation. 2.1 ml of the diluted samples was concentrated by ultracentrifugation at 20,000 g, 4°C for 2 hours. After ultracentrifugation, 1.9 ml of the supernatant was removed, and the remaining 200 *μ*l was left for nucleic acid extraction.

### 2.4. RNA Extraction, PCR, and Sanger Sequencing

Viral RNA was extracted from 200 *μ*l of plasma samples using the Viral RNA Extraction Kit (Guangzhou Life Technologies Daan Diagnostics Co., Ltd.) according to the manufacturer's instructions. A validated Sanger sequencing method amplifies the entire pol gene containing the reverse transcriptase, protease, and integrase region (Guangzhou Life Technologies Daan Diagnostics Co., Ltd.). The positive PCR products were purified, and the PCR products were sequenced using the 3500XL DX Genetic Analyzer [16]. Subsequently, HIV-1 DRMs and resistance interpretations were determined using the Stanford HIVdb algorithm version 9.0. HIV-1 subtypes and CRFs were defined using the COMET online tool (http://comet.retrovirology.lu). The phylogenetic tree was constructed using MEGA (Version 7.0, USA) software, and the bootstrapping test was performed.

### 2.5. Statistical Analysis

Continuous variables were described as the median and interquartile range (IQR), while percentages presented categorical variables. HIV-1 genotyping was successful when both amplification and DNA sequencing were successful. The overall success rate of LCR was compared with ultracentrifugation. Furthermore, the success rates at distinct VL categories of the two methods were also calculated. Drug resistance mutations of diluted samples were compared with those of undiluted samples. Statistical analysis was performed using GraphPad 7 (GraphPad Software, La Jolla, CA, USA).

## 3. Results

### 3.1. Patient Characteristics

Twenty participants were included, and their plasma samples were serially diluted to 1000 copies/ml, 400 copies/ml, 200 copies/ml, and 100 copies/ml, respectively. 95% of the participants (19/20) were male, and the route of transmission of 18 patients was through homosexual contact. The median VL of undiluted samples was 33874 copies/ml (IQR: 29197-37217 copies/ml). Of the 20 participants enrolled, CRF_01 AE accounted for 45%, and subtypes CRF07_BC, B, C, and CRF55_01B accounted for 25%, 15%, 5%, and 10%, respectively. The characteristics of the 20 participants are shown in Supplementary Table 1.

### 3.2. Comparison of the Overall Success Rate of Two Concentration Methods

**Figure 1:**
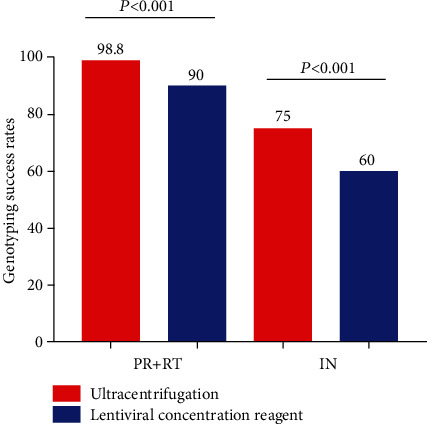
Compare the overall success rate of two different concentration methods.

When samples were concentrated by ultracentrifugation, the overall success rate was 98.8% (79/80) in the PR and RT regions and 75% (60/80) in the IN region. When enriched with LCR, the total success rate of PR and RT regions was 90% (72/80), and the complete success rate of the IN region was 60% (48/80). The overall success rate of ultracentrifugation was higher than LCR, and the difference was statistically significant (PR and RT: *P* < 0.001; IN: *P* < 0.001). The results are shown in Figure 1.

### 3.3. Comparison of the Success Rates at Different VL Categories

**Figure 2:**
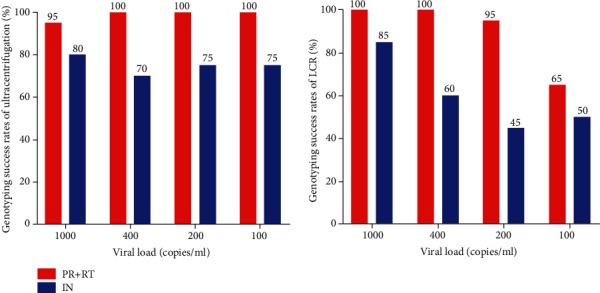
Compare the success rates of different VL categories by two distinct concentration methods.

Using ultracentrifugation, when HIV RNA was 1000 copies/ml, 400 copies/ml, 200 copies/ml, and 100 copies/ml, the success rates of PR and RT regions were 95%, 100%, 100%, and 100%, respectively, whereas the success rates in the IN region were 80%, 70%, 75%, and 75%, respectively. Using LCR, when HIV RNA was 1000 copies/ml, 400 copies/ml, 200 copies/ml, and 100 copies/ml, the success rates in PR and RT regions were 100%, 100%, 95%, and 65%, respectively, whereas the success rates in the IN region were 85%, 60%, 45%, and 50%, respectively. The results are shown in Figure 2.

### 3.4. Compared DRMs with Undiluted Samples by Two Methods at Different Viral Loads

Supplementary table 2 shows the resistance profiles of undiluted clinical specimens and is used as a reference to assess the detection of additional mutations or lack of mutations. In our study, we found that sample DR-316B4 missed the E138G mutation and DR-217B3 detected K219KE mutation, which was not detected in other samples. Mutations in other specimens were consistent with undiluted samples by using ultracentrifugation. The results are shown in Table 1. Furthermore, we found that the DR-387A2 missed the E138A mutation, and mutations in other specimens were consistent with undiluted samples using LCR. The results are shown in Table 2.

### 3.5. Phylogenetic Tree Analysis of Two Concentration Methods

**Figure 3:**
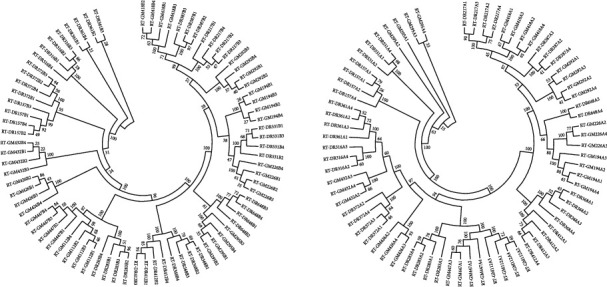
Gene evolution analysis of serial dilution samples of the two concentration methods.

Phylogenetic tree analysis of LCR and ultracentrifugation was performed to evaluate the evolutionary relationship of diluted samples. Sequences for each dilution series are closely related and support reproducible scenarios for low VL sequencing. Gene evolution analysis of serial dilution samples using ultracentrifugation and LCR is shown in Figure 3.

## 4. Discussion

To our knowledge, this is the first study in China to explore the feasibility and applicability of LCR in LLV samples. We offer a potentially attractive approach to concentrating the HIV-1 virus. Although the enrichment effect of the LCR was slightly inferior to ultracentrifugation, the difference was statistically significant (PR and RT: *P* < 0.001; IN: *P* < 0.001). The success rate of LCR in PR and RT regions can achieve 95% when the VL ≥ 200 copies/ml. However, without LCR or ultracentrifugation, the success rate is only about 30%.

According to the “*U* = *U*” concept of the Prevention Access Campaign in the United States, HIV-1 infected patients with VL consistently below 200 copies/ml have a negligible risk of transmitting HIV [17]. Also, compared with VL of more than 200 copies/ml, previously published studies have shown that LLV with VL of 50–200 copies/ml has a relatively lower risk of VF [5, 6]. In our research, the LCR has good applicability and effectiveness for VL of more than 200 copies/ml and can be used as an effective alternative for laboratories that cannot afford an ultracentrifuge.

A worrisome problem with HIV RNA sequencing in LLV patients is the selection and misrepresentation of the virological profile in vivo [18]. We found that the DR-387A2 missed the E138A mutation, and the DR-316B4 missed the E138G mutation, which may be due to uneven dilution or the frequency of mutant virus strains being less than 20% after dilution. At the same time, the limitation of SS, which has weak sensitivity in detecting DRMs in <20% virus population, is also emphasized [19, 20]. In addition, the K219KE mutation was detected in DR-217B3, a mixed mutation not seen in other diluted samples. A closer analysis of the peak diagram showed that this mixed mutation exists in this sample. In DR-217B3, the G peaks of three sequencing primers were higher than the 20% peak cut-off for detection by sequencing analysis. However, in DR-217B2 and DR-217B4, only one sequencing primer had a G peak higher than 20%. However, none of the three sequencing primers showed a G peak in DR-217B1. This difference may be caused by the heterogeneity of PCR amplification, uneven dilution, and the limitation of Sanger sequencing.

Appropriate management of patients with HIV-1 infection requires periodic assessment of HIV-1 viremia and monitoring of drug resistance mutations. The current guidelines oversight and do not guide LLV patients. Hence, managing LLV patients is complicated and controversial [21–23]. Furthermore, without the data of RGT, clinicians can maintain the regimen when DRMs have already emerged or switch to a new regimen empirically when the virus is still sensitive.

Most laboratories use ultracentrifugation to concentrate the HIV-1 virus, which implies using expensive equipment. In addition, there are gaps with foreign countries in machine intelligence, temperature control, and other aspects. In our study, LCR could be applied combined with the in-house SS method for monitoring DRMs in HIV-1 patients with LLV. The LCR assay would be a helpful tool to concentrate the HIV-1 virus. The LCR assay costs only $ 2.7 for one sample compared to ultracentrifugation, which costs approximately $ 2837 to $ 11350. This assay will facilitate surveillance studies and support drug resistance testing for LLV samples in small- or mid-sized laboratories.

There were some limitations in this study. Firstly, the amplification efficiency for the IN region was significantly lower than that for the PR and RT regions. This phenomenon not only exists in LLV samples but also in samples with high VL at baseline or treatment failure, which may be related to primer specificity [24]. The primer design for the IN region is still not mature and needs to be further optimized in the future. Secondly, the LCR requires an input volume of 2.1 ml to concentrate samples with <1000 copies/ml, which is significantly more than the standard 200-500 *μ*l for samples with ≥1000 copies/ml.

In summary, ultracentrifugation can be purchased in laboratories with good economic conditions, and LCR can be purchased in areas with poor economic conditions. The two effective concentration methods could support drug resistance testing of HIV-1 LLV samples.

## 5. Conclusion

In summary, LCR will provide strong support for monitoring DRMs in LLV samples of HIV-1 patients and can be an effective alternative for small- and medium-sized laboratories that cannot afford an ultracentrifuge.

## Figures and Tables

**Table 1:** Samples with different DRMs compared with undiluted samples by the ultracentrifugation method.

ID card	Subtype	PI	NRTI	NNRTI
DR-217B1	CRF01_AE	None	A62V, K65R, M184V	Y181C, G190S
DR-217B2	CRF01_AE	None	A62V, K65R, M184V	Y181C, G190S
DR-217B3	CRF01_AE	None	A62AV, K65R, M184V, K219KE	Y181C, G190S
DR-217B4	CRF01_AE	None	A62AV, K65R, M184V	Y181C, G190S
DR-316B1	CRF07_BC	None	None	E138G
DR-316B2	CRF07_BC	None	None	E138G
DR-316B3	CRF07_BC	None	None	E138G
DR-316B4	CRF07_BC	None	None	None

Abbreviations: NRTI: nucleoside reverse transcriptase inhibitors; NNRTI: nonnucleoside reverse transcriptase inhibitor; PI: protease inhibitor.

**Table 2:** Samples with different DRMs compared with undiluted samples by the LCR method.

ID card	Subtype	PI	NRTI	NNRTI
DR-387A2	CRF01_AE	M46I	None	None
DR-387A3	CRF01_AE	M46I	None	E138A
DR-387A4	CRF01_AE	M46I	None	E138A

Abbreviations: NRTI: nucleoside reverse transcriptase inhibitors; NNRTI: nonnucleoside reverse transcriptase inhibitor; PI: protease inhibitor; LCR: lentivirus concentration reagent.

## Data Availability

Part of the data had been presented at the Fifth National Academic Conference on infectious diseases in China, in May 2022.

## Supplementary Materials

Supplementary Materials The characteristics of the 20 participants are shown in Supplementary Table 1. Supplementary table 2 shows the resistance profiles of undiluted clinical specimens and is used as a reference to assess the detection of additional mutations or lack of mutations.
