# Supplementary material for: A Concentration Method for HIV Drug Resistance Testing in Low-Level Viremia Samples
Source: Biomed Res Int. 2022 Nov 23;2022:2100254. doi: 10.1155/2022/2100254 (PMC9711986; doi:10.1155/2022/2100254)
Supplement: Supplementary Materials — The characteristics of the 20 participants are shown in Supplementary Table 1. Supplementary table 2 shows the resistance profiles of undiluted clinical specimens and is used as a reference to assess the detection of additional mutations or lack of mutations. [file 2100254.f1.zip › Supplementart Table 2.docx]

**Supplementary Table 2** Resistance profiles of undiluted clinical specimens

| **Case ID** | **Sex** | **Age** | **VL(cps/ml)** | **Subtype** | **Drug-resistant mutation** | | | |
| --- | --- | --- | --- | --- | --- | --- | --- | --- |
|  |  |  |  |  | **NNRTIs** | **NRTIs** | **PIs** | **INSTIs** |
| DR-157 | M | 47 | 35017 | CRF07_BC | V106VI | - | - | - |
| DR-217 | M | 39 | 27546 | CRF01_AE | Y181C, G190S | A62V,K65R,M184V | - | - |
| DR-283 | M | 57 | 36367 | B | V106I | - | - | - |
| DR-316 | M | 65 | 49913 | CRF07_BC | E138G | - | - | - |
| DR-361 | M | 20 | 22767 | CRF07_BC | V179D | - | - | - |
| DR-368 | F | 27 | 32731 | CRF55_01B | V179E | - | - | - |
| DR-372 | M | 39 | 31582 | CRF07_BC | E138G | - | - | - |
| DR-387 | M | 26 | 29192 | CRF01_AE | E138EA | - | M46I | - |
| DR-412 | M | 46 | 38861 | CRF55_01B | K103N,E138G,V179E | - | - | - |
| DR-531 | M | 36 | 25588 | CRF01_AE | V106M,K103R,V179D | M184V | M46ML | - |
| DR-648 | M | 40 | 29469 | CRF01_AE | V179D | - | - | - |
| GM-112 | M | 29 | 21129 | B | V106I | - | - | - |
| GM-194 | M | 19 | 29610 | CRF01_AE | P225H,G190A | - | - | - |
| GM-226 | M | 26 | 37501 | CRF01_AE | Y181C | - | - | - |
| GM-292 | M | 48 | 35904 | CRF01_AE | V179E | - | - | - |
| GM-295 | M | 42 | 35425 | CRF01_AE | V179D | - | - | - |
| GM-418 | M | 34 | 29213 | CRF01_AE | V179D | - | - | - |
| GM-426 | M | 29 | 47816 | C | V179D | - | - | - |
| GM-432 | F | 55 | 42346 | CRF07_BC | K103N | - | - | - |
| GM-447 | M | 24 | 35770 | B | V106I | - | - | - |
