# Supplementary material for: A Concentration Method for HIV Drug Resistance Testing in Low-Level Viremia Samples
Source: Biomed Res Int. 2022 Nov 23;2022:2100254. doi: 10.1155/2022/2100254 (PMC9711986; doi:10.1155/2022/2100254)
Supplement: Supplementary Materials — The characteristics of the 20 participants are shown in Supplementary Table 1. Supplementary table 2 shows the resistance profiles of undiluted clinical specimens and is used as a reference to assess the detection of additional mutations or lack of mutations. [file 2100254.f1.zip › Supplementary Table 1.docx]

**Supplementary Table 1** Characteristics of the study participants

| **Variable** | **Patients (n=20)** |
| --- | --- |
| Age (years) |  |
| <30 | 8(40%) |
| 30-50 | 10(50%) |
| >50 | 2(10%) |
| Gender |  |
| Male | 19(95%) |
| Female | 1(5%) |
| HIV transmission route |  |
| Homosexual | 18(90%) |
| Heterosexual | 2(10%) |
| Plasma viral load, copies/mL |  |
| 20 000 -30 000 | 8(40%) |
| 30 001 – 40 000 | 9(45%) |
| 40 001 – 50 000 | 3(15%) |
| Subtype |  |
| CRF01_AE | 9(45%) |
| CRF07_BC | 5(25%) |
| B | 3(15%) |
| C | 1(5%) |
| CRF55_01B | 2(10%) |
